# *PGL*, encoding chlorophyllide a oxygenase 1, impacts leaf senescence and indirectly affects grain yield and quality in rice

**DOI:** 10.1093/jxb/erv529

**Published:** 2015-12-25

**Authors:** Yaolong Yang, Jie Xu, Lichao Huang, Yujia Leng, Liping Dai, Yuchun Rao, Long Chen, Yuqiong Wang, Zhengjun Tu, Jiang Hu, Deyong Ren, Guangheng Zhang, Li Zhu, Longbiao Guo, Qian Qian, Dali Zeng

**Affiliations:** ^1^State Key Laboratory for Rice Biology, China National Rice Research Institute, Hangzhou 310006, Zhejiang, China; ^2^Key Laboratory of Crop Physiology, Ecology and Genetic Breeding, Ministry of Education, Jiangxi Agricultural University, Nanchang 330045, Jiangxi, China

**Keywords:** Chlorophyll b, chlorophyllide a oxygenase, leaf senescence, pale green leaf, rice.

## Abstract

Chlorophyll (Chl) b is a ubiquitous accessory pigment in land plants, green algae, and prochlorophytes. This pigment is synthesized from Chl a by chlorophyllide a oxygenase and plays a key role in adaptation to various environments. This study characterizes a rice mutant, *pale green leaf* (*pgl*), and isolates the gene *PGL* by using a map-based cloning approach. *PGL*, encoding chlorophyllide a oxygenase 1, is mainly expressed in the chlorenchyma and activated in the light-dependent Chl synthesis process. Compared with wild-type plants, *pgl* exhibits a lower Chl content with a reduced and disorderly thylakoid ultrastructure, which decreases the photosynthesis rate and results in reduced grain yield and quality. In addition, *pgl* exhibits premature senescence in both natural and dark-induced conditions and more severe Chl degradation and reactive oxygen species accumulation than does the wild-type. Moreover, *pgl* is sensitive to heat stress.

## Highlight

*PGL* encodes CAO1 in rice, which is essential for Chl b synthesis and affects Chl synthesis and degradation. *PGL* also impacts leaf senescence and indirectly affects grain yield and quality.

## Introduction

Chlorophyll (Chl) is a green pigment found in cyanobacteria and the chloroplasts of algae and plants. Chl is an essential component of photosynthetic apparatuses and plays critical roles in plant development (Zhen *et al.*, 2014). Aside from providing green color, Chl is also extremely important in photosynthesis because it plays essential roles in harvesting light energy and converting it to chemical energy (Fromme *et al.*, 2003). Increasing the Chl content in rice is regarded as an important approach to enhancing the photosynthesis rate (Huang *et al.*, 2013) to drive the accumulation of more photo-assimilates and ultimately increase crop yield (Bansal *et al.*, 1999; Mitchell and Sheehy, 2006). Hence, breeders strive to develop plants with a long stay-green period to increase the yield potential of rice.

Two Chl species are present in higher plants: Chl a and Chl b. Most Chls are assembled with apoproteins to form Chl-protein complexes in plant leaves. Chl a is a component of both photosynthetic reaction centers and light-harvesting Chl-protein complexes (LHC), whereas Chl b only exists in LHC (Green and Durnford, 1996; Liu *et al.*, 2004). The chemical structures of the two main Chl pigments differ in one side chain of the tetrapyrrole. Chl a has a methyl group, whereas Chl b carries a formyl group at the corresponding position in ring B. Moreover, their absorption spectra are different. Chl b containing-antenna complexes can harvest light energy at ~470 and 650nm; light at this wavelength is not efficiently absorbed by Chl a. Therefore, organisms that use Chl b in their LHC can harvest a wider range of light energy than those that are limited to Chl a. Aside from its light-harvesting function, Chl b is also important for controlling photosynthetic antenna size by regulating the stability of the LHC (Bellemare *et al.*, 1982). LHCII levels increase when Chl b synthesis is activated by the Chl precursor and decrease in Chl b-less/deficient mutants (Paulsen *et al.*, 1993; Espineda *et al.*, 1999; Bailey *et al.*, 2001). The binding of Chl b to the LHC proteins stabilizes the latter in the thylakoid membranes (Paulsen *et al.*, 1993; Lindahl *et al.*, 1995). By contrast, LHC proteins without Chl b binding are degraded by proteases (Hoober and Eggink, 2001).

Chl loss and disassembly of the photosynthetic apparatus are the most remarkable events in leaf senescence (Buchanan-Wollaston, 1997). A delay in Chl degradation can result in the stay-green phenotype, which shows a slower progression of senescence compared with a standard reference (Park *et al.*, 2007). Senescence can be regarded as an oxidative process because of the overproduction of reactive oxygen species (ROS), such as hydrogen peroxide and superoxide radicals (Piquery *et al.*, 2000). ROS represent a ubiquitous signal and possible co-stressor of environment conditions including heat stress (Tomanek, 2014). The increased ROS levels under stresses can result in oxidative damage to cellular constituents and ultimately cell death (Mittler, 2002). Heat stress causes premature leaf senescence. Heat-induced leaf senescence is characterized by the loss of Chl and proteins, weakened metabolic activities, and oxidative damage (Wahid *et al.*, 2007).

The Chl synthesis pathway has been well characterized, and the genes encoding enzymes involved in Chl synthesis have been isolated (Beale, 2005; Nagata *et al.*, 2005). In the last step of Chl biosynthesis, Chl b is synthesized from Chl a through the oxidation of a methyl group on the D ring, which is catalyzed by chlorophyllide a oxygenase (CAO) (Tanaka *et al.*, 1998; Espineda *et al.*, 1999). The *CAO* gene was first identified in *Chlamydomonas reinhardtii* with six Chl b-less mutants (Tanaka *et al.*, 1998). The gene’s sequence is highly conserved from cyanobacteria to higher plants (Tomitani *et al.*, 1999; Mueller *et al.*, 2012). CAO is a mononuclear iron-containing protein and has a [2Fe–2S] Rieske center and a tyrosine radical (Tanaka *et al.*, 1998; Eggink *et al.*, 2004). CAO contains three domains, which are sequentially named from the N terminus as the A, B and C domains (Nagata *et al.*, 2004). The C domain is the conserved core domain of CAO, which contains a Rieske center and non-heme iron-binding motifs and catalyzes the conversion of Chl a to Chl b (Tomitani *et al.*, 1999; Nagata *et al.*, 2004). The B domain is less conserved and may function as a link that stabilizes CAO (Sakuraba *et al.*, 2007). The A domain is thought to play a role in the regulation of the CAO protein level. Interestingly, this regulatory mechanism does not operate when the Chl b synthesizing activity is deficient (Yamasato *et al.*, 2005, 2008). Another study demonstrated that a sequence with ten amino acids is essential for CAO degradation. This sequence functions as the CAO degron for a chloroplast protease (Sakuraba *et al.*, 2009). Chl b synthesis is known to be critical for LHC formation. However, if Chl b is over-produced, the accumulated free Chl b induces photodamage. Therefore, regulating CAO activity is important for Chl synthesis and chloroplast development.

Two homologous genes of *CAO, OsCAO1* and *OsCAO2*, have been identified from the rice genome. These genes are highly homologous and positioned in tandem, which is most likely due to recent gene duplications (Lee *et al.*, 2005). However, their expression patterns are entirely different. *OsCAO1* is expressed in green tissues, with a higher expression level during the daytime and a lower expression level in the late afternoon and at night. In contrast, *OsCAO2* functions in non-photosynthetic tissues, and its expression increases after dusk and decreases after the dark phase ends. These findings indicate that *OsCAO1* and *OsCAO2* play different roles in rice development. *OsCAO1* plays a major role in Chl b synthesis and chloroplast development under the light, whereas *OsCAO2* may function in the dark. A study of the two T-DNA mutants of *OsCAO1* and *OsCAO2* found that *OsCAO2* knockout mutant leaves do not differ significantly from wild type leaves, whereas *OsCAO1* knockout mutants have pale green leaves. These findings suggested that *OsCAO2* cannot compensate for the loss of *OsCAO1* (Lee *et al.*, 2005).

This study performs a map-based cloning of the *pale green leaf* (*pgl*) locus in rice (*Oryza sativa*) and reveals that *pgl* harbors a single-base substitution in the coding region of *OsCAO1*, which results in a premature translational termination. On the basis of the expression analysis of *PGL* and related genes and the phenotypic characterization of *pgl*, the gene product of *PGL* is proposed to play important roles in Chl b and Chl a syntheses, Chl degradation, and leaf senescence in rice.

## Materials and methods

### Plant materials and growth conditions

The *pgl* mutant was derived from an M2 population of the *japonica* rice variety Yunyin (YY) by ethyl methane sulphonate (EMS) mutagenesis as previously described (Guo *et al.*, 2005). The *japonica* variety YY and the *indica* variety TN1 were used to segregate the population construction. The plants grown under natural conditions were grown in a paddy field at the China National Rice Research Institute (CNRRI), Fuyang, Zhejiang Province, China and Lingshui, Hainan Province, China. The plants used in the heat stress and low light experiments were grown in a growth chamber. The chamber conditions for heat stress were as follows: the rice plants were treated at 42°C for 16h during the daytime and 35°C for 8h at night with a 32/25°C (day/night) temperature regime as a control. The chamber conditions for the low light experiment were as follows: the rice plants were grown under low (30 μmol m^−2^ s^−1^) or moderate (150 μmol m^−2^ s^−1^) light conditions at 32°C, followed by 8h dark at 28°C.

### Chl content and rice quality determination

The total Chl in the leaves was extracted with 80% acetone. The extract was analyzed using a spectrophotometer (Shimadzu UV2400, Japan). The total Chl, Chl a, and Chl b contents were estimated with light absorption values of 470, 645 and 663nm, respectively, according to Porra *et al.* (1994). The rice quality traits were measured as previously described (Su *et al.*, 2011).

### Transmission electron microscope (TEM) analysis

The leaf samples for the TEM analysis were harvested from 4-week-old plants, 20 d after flowering in the paddy. The detached leaves were soaked in a fixation buffer (2.5% glutaraldehyde in 100mM phosphate buffer, pH 7.4). The polymerization and staining of the leaf samples were based on the method of Tanaka *et al.* (2003). The prepared samples were observed under a Hitachi H-7650 (Japan) TEM.

### Map-based cloning of *PGL*

A mapping population was derived from a cross between *pgl* and TN1. A total of 2207 individuals with the mutant phenotype were used for mapping. The initial localization was determined with a total of 163 simple sequence repeat (SSR) markers scattered among all 12 chromosomes (www.gramene.org). Accordingly, 296 individuals were used for the primary mapping of *PGL*. For further mapping, new sequence tagged site (STS) and SSR markers between the two flanking markers were designed based on the differences between the genomic DNA sequences of *japonica* variety Nipponbare and *indica* variety 9311 (www.gramene.org/resources). All of the PCR products were separated on 4–5% agarose gels for visualization.

### Rice transformation

For the complementation of the *pgl* mutation, a 6959bp genomic DNA fragment containing the *PGL* coding region along with the upstream and downstream sequences was cloned into the binary vector pCAMBIA1300 to generate the transformation construct, *pCAO1F*. The binary construct was introduced into the calli generated from the mature seed embryos of *pgl* through the *Agrobacterium* (EHA105)-mediated method. Both the sense and anti-sense of *PGL* coding sequences (CDS) were inserted into the binary vector pHQSN containing the 35S promoter (*p35S::PGL* and *p35S::anti-PGL*) and introduced into YY to overexpress and knock-down *PGL*, respectively, thereby allowing further exploration of the functions of *PGL* in rice. Rice transformation was performed as previously described (Li and Li, 2003).

### Subcellular localization of PGL

To investigate the subcellular localization of PGL, the coding region sequence of *PGL* without the termination codon was cloned into the pCaMV35S-GFP binary vector to fuse PGL and the enhanced green fluorescent protein (eGFP). The fusion constructs (*p35S::PGL-GFP*) and the control (*p35S::GFP*) were transformed into tobacco (*Nicotiana benthamiana*) epidermal leaf cells using *Agrobacterium*-mediated injection. The cells were then examined under a confocal fluorescence microscope (Leica TCS SP5, Germany) after 48h of incubation. The GFP and the Chl fluorescence were recorded at 522 and 680nm, respectively. A truncated PGL was fused to the eGFP (*p35S::OsCAO1* ^*pgl*^ *-GFP*) to test whether the premature termination of PGL in *pgl* affects the protein’s localization and was subsequently transformed into tobacco for GFP detection.

### Analysis of reactive oxygen species

The accumulation of the superoxide anion was monitored with nitroblue tetrazolium (NBT) (0.5mg ml^−1^ in 10mM potassium phosphate buffer, pH 7.6). Hyperoxide was detected by 3,3′-diaminobenzidin (DAB, 1mg ml^−1^ in 50mM Tris acetate buffer, pH 5.8). The staining and bleaching of the samples were performed as previously described (Wang *et al.*, 2013).

### RNA extraction and quantitative real-time PCR analysis

Total RNA was extracted from rice tissues using a Total RNA Extraction Kit (Axygen, cat NO, AP-MN-MS-RNA-250, USA). All of the samples were treated with DNase I (Promega; www.promega.com, USA). First-strand cDNA synthesis was primed with an oligo(dT) primer by using a ReverTra Ace qPCR-RT kit (Toyobo, Japan). Quantitative real-time PCR (qRT-PCR) was performed using a 2×SYBR Green PCR Master Mix (Applied Biosystems, USA) in an Applied Biosystems 7900HT Real-time PCR System. The mRNA expression of these genes were quantified, including *OsCAO1*, *OsHEMA*, *OsCHLH*, *OsPORA*, *OsPORB*, *OsDVR*, *OsYGL1*, *OsLhcb1*, *OsLhcb4*, *OsNOL*, *OsNYC1*, *OsNYC3*, *OsNYC4*, *OsPAO*, *OsNCCR1*, *OsCatB*, *OsPOD1*, *OsPOD2*, *OsAPX1*, *OsAPX2*, *OsH36* and *Actin1*. The transcript data were normalized using *Actin1* as an internal control. The error bars indicate the standard error of the mean. All of the experiments were performed in triplicate.

## Results

### The phenotype of *pgl*

**Fig. 1.**
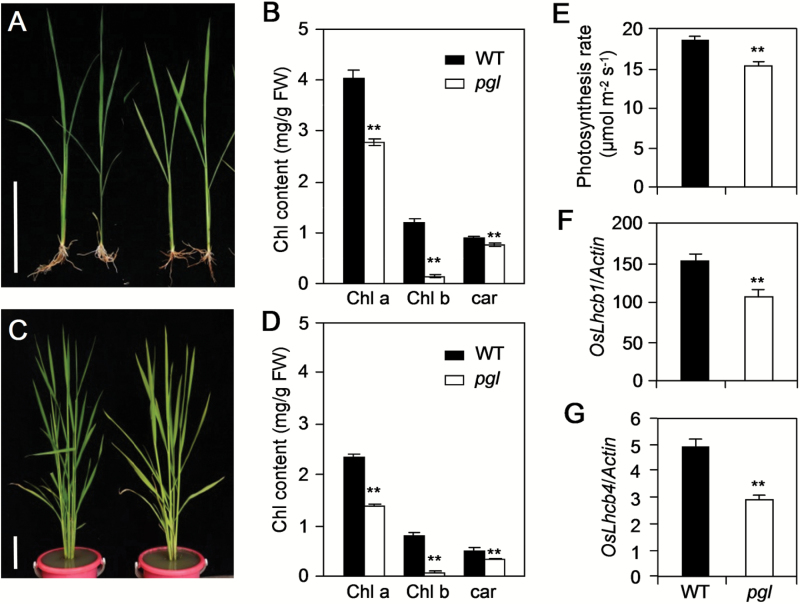
Phenotypic characterization of *pgl*. (A, C) Phenotypes of WT (left) and *pgl* (right) at 14 and 80 d after sowing (DAS). Bar, 10cm. (B, D) Chl contents including Chl a, Chl b and carotene in WT and *pgl* plants at 14 DAS (B) and 80 DAS (D). (E) Photosynthesis rate of WT and *pgl* plants at the filling stage. (F, G) Expression levels of *Lhcb1* and *Lhcb4* in WT and *pgl* plant at the heading stage. *Lhcb1*, light-harvesting Chl a/b-binding protein of PS II (LOC_Os10g41780); *Lhcb4* (LOC_Os07g37240). Mean and SD values were obtained from three biological replicates. *, *P*<0.05; **, *P*<0.01 (Student’s *t*-test).

A *pgl* mutant was identified in the mutagenized population to further understand the genetic mechanism of pigments. The *pgl* mutant exhibited pale-green leaves compared with the wild-type (WT) throughout the entire developmental process both in Fuyang, Zhejiang Province (120°00′E, 30°06′N) and Lingshui, Hainan Province (110°02′E, 18°03′N). *pgl* showed a more obvious phenotype at the tillering stage than at the seedling stage (Fig. 1A, C). The Chl content analysis indicated that all three Chl species contents were decreased in *pgl* compared with WT, especially Chl a and Chl b (Fig. 1B, D). In *pgl*, the ratio of Chl a/Chl b was dramatically increased compared with that of WT, reaching 21.4 and 27.7 at the seedling and tillering stages, respectively, because almost no Chl b could be detected in *pgl*. The ratio of Chl a/Chl b was 3.38 and 2.97 in WT rice at the seedling and tillering stage, respectively (Supplementary Table S1 available at *JXB* online).

Chl is one of the most important players in photosynthesis. The photosynthesis in the flag leaves at the heading stage was measured to investigate whether the massive decrease of the Chl content affected photosynthesis. As expected, the photosynthesis rate was significantly lower in *pgl* than in WT (Fig. 1E). Light-harvesting chlorophyll-binding proteins make up light-harvesting complex II for light harvesting, which was closely related to photosynthesis rate (Caffarri *et al.*, 2004). The expression levels of the photosynthesis-associated genes (i.e. *Lhcb1* and *Lhcb4*) were also examined in the WT and *pgl* plants. The results showed that *OsLhcb1* and *OsLhcb4* were strongly down-regulated in *pgl* compared with the WT (Fig. 1F, G). The *pgl* phenotypic characterization suggests that *PGL* is essential for photosynthesis and Chl synthesis, especially Chl b synthesis.

### *pgl* shows reduced grain yield and quality

**Fig. 2.**
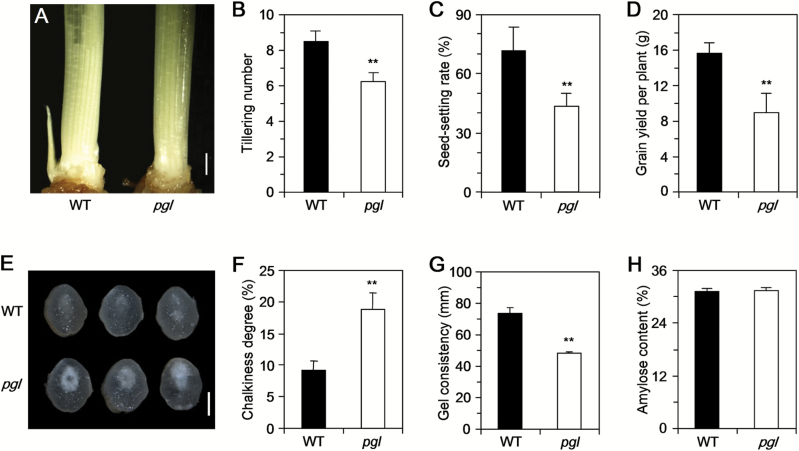
*pgl* affects rice yield and quality characteristics. (A) Phenotype of WT (left) and *pgl* (right) 15 d after flowering. Bar, 20cm. (E) Serious degree of chalkiness in *pgl*. Bar, 1mm. (B–D, F–H) Comparison of yield trait and quality characteristics: tillering number (B); seed-setting rate (C); grain yield per plant (D); chalkiness degree (F); gel consistency (G); amylose content (H). *, *P*<0.05; **, *P*<0.01 (Student’s *t*-test).

A comparative analysis of the agronomic traits of WT and *pgl* was performed due to the substantially lower photosynthesis rate observed in *pgl* (Fig. 2; Supplementary Table S2). *pgl* plants demonstrated a lower tiller number and seed-setting rate than that of the WT plants (Fig. 2A–C). The seed-setting rate was only 43.2% in *pgl*, whereas it reached up to 71.0% in the WT. However, no significant difference between *pgl* and WT was found with respect to plant height, branch, grain numbers or grain weight (Supplementary Table S2). Consequently, the grain yield per plant for *pgl* was 8.92g. This value was ~57% of the 15.60g measured in WT (Fig. 2D; Supplementary Table S2).

The amount of photosynthesis occurring during the grain-filling stage is an important physiological factor that affects biomass and grain yield and further influences rice quality (Lin *et al.*, 2002; Baig *et al.*, 2005; Feng *et al.*, 2013). Grain qualities including chalkiness degree (CD), gel consistency (GC), amylose content (AC) and gelatinization temperature (GT) in *pgl* and WT were further investigated (Fig. 2E–H; Supplementary Table S3). The CD in *pgl* was higher than that in WT (Fig. 2E, F). Moreover, the GC in *pgl* was lower than that in the WT (Fig. 2G). Nevertheless, no significant difference was found in the AC or GT between *pgl* and WT (Fig. 2H; Supplementary Table S3). In summary, the *PGL* mutation resulted in the reduction of grain yield and quality in *pgl*.

### Map-based cloning of *PGL*

**Fig. 3.**
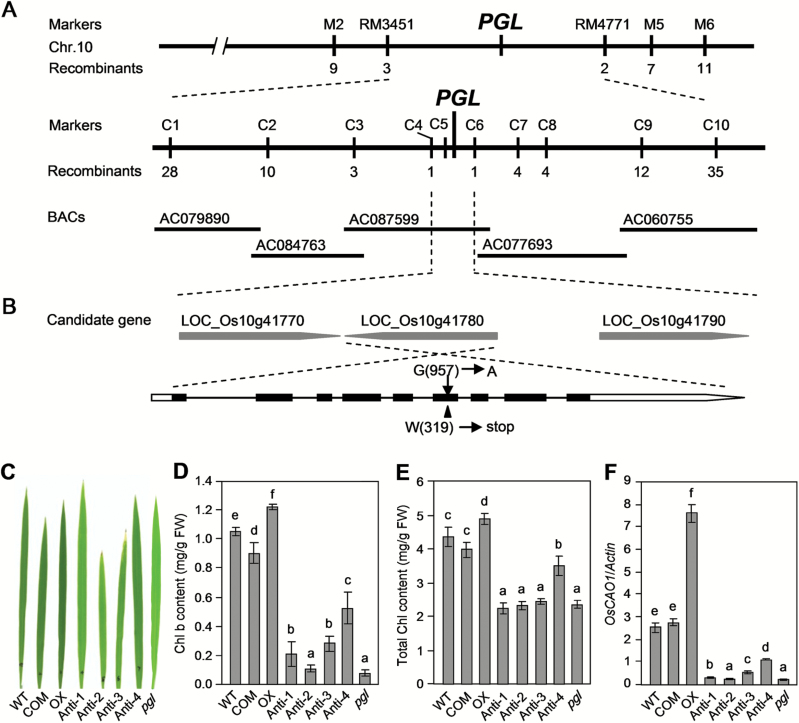
Map-based cloning and identification of the *PGL* gene. (A) The gene was mapped to the interval between molecular markers RM3451 and RM4771 on chromosome 10 and further delimited to a ~35kb genomic region between markers C4 and C6; the numbers below the markers indicate the number of recombinants. (B) The candidate region contained three ORFs. The *pgl* mutation is shown at position 957 in LOC_Os10g35370 and changes W at position 319 to a stop code. The arrow indicates the mutation point. (C) The leaf color of WT and transgenic plants. (D) The Chl b content and (E) total Chl content of flag leaf in WT and transgenic plants. (F) The *PGL* expression levels as detected by qRT-PCR in WT and transgenic plants. WT, wild-type; com, *pCAO1F*; OX, *p35S::PGL*; Anti, *p35S::anti-PGL*. Different letters indicate a significant difference at the 1% level (Duncan’s multiple range test).

A map-based cloning approach was used to isolate *PGL* and investigate the molecular basis of the *pgl* phenotype. Accordingly, 2207 plants with the *pgl* phenotype were selected from the population generated by crossing *pgl* with TN1 (i.e. 8943 plants). The ratio of individuals with a normal phenotype to those with a *pgl* phenotype showed a good fit to the expected value [i.e. 3:1 (χ^2^=0.4929, *P*=0.4826)]. The reciprocal crosses between *pgl* and *indica* varieties (i.e. ZF802, NJ06 and 9311) were performed to confirm the segregation ratio. All of the F_1_ hybrids showed a normal leaf color. The segregation of the normal individuals to *pgl* phenotype plants in the F_2_ population also showed a good fit to 3:1 (Supplementary Table S4). The results indicate that *pgl* is controlled by a single recessive gene. A bulked segregant analysis was employed to perform the preliminary mapping. A total of 163 pairs of SSR markers evenly distributed in the rice genome were selected for the analysis of the SSR polymorphisms between *pgl* and TN1. The identified polymorphic SSR markers were used to detect polymorphisms between the normal and mutant DNA pools, which were derived from the progeny of ‘*pgl*/TN1.’ Subsequently, *PGL* was mapped between SSR markers RM3451 and RM4771 on chromosome 10 (Fig. 3A).

A set of polymerase chain reaction (PCR)-based molecular markers was developed for fine mapping of *PGL*. *PGL* was further narrowed to an interval of ~35kb between markers C4 and C6, which contains three predicted ORFs (Fig. 3A, B; Supplementary Table S5). The 35-kb genomic DNA segments from the WT and *pgl* plants were sequenced and compared to determine the mutation in *pgl*. A mutation was identified in the predicted LOC_Os10g41780 gene in the *pgl* genome, which encodes chlorophyllide a oxygenase (CAO1). This gene had been previously reported by Lee *et al.* (2005). In the remainder of this paper, *pgl* will continue to be used as the mutant line name and *PGL* or *OsCAO1* as the gene name. A comparison of the LOC_Os10g41780 CDS between the *pgl* and WT plants showed that the mutation in *pgl* resulted from a substitution of an A for G (G957→A957) in the sixth exon, leading to a non-synonymous change from tryptophan (W) to a stop code and thereby resulting in the premature termination of translation (Fig. 3B).

A genetic complementation experiment was conducted to confirm that the *PGL* mutation was responsible for the mutant phenotype. Supplementary Table S6 illustrates the primers used for the vector construction in this study. A complementation vector containing the entire *PGL* coding region, a 2125-bp upstream region and a 1030-bp downstream sequence was inserted into the binary vector pCAMBIA1300 (*pCAO1F*, COM). The constructed and empty vectors were introduced into the mutant. The pale-green leaves were completely restored to normal color in the transgenic plants (Fig. 3C). The Chl contents were also restored to normal (Fig. 3D, E). These results demonstrated that the cloned candidate gene *OsCAO1* was indeed responsible for the *pgl* phenotype.

Accordingly, *PGL* was overexpressed using the 35S cauliflower mosaic virus promoter (*35S::PGL*, OX) in WT to further investigate the function of *PGL* in plant development. An expression vector containing the anti-sense *PGL* fragment was also constructed to knock-down *PGL* in WT plants (*35S::anti-PGL*, Anti). The OX plants displayed a normal leaf color, whereas the Anti lines showed pale-green leaves (Fig. 3C). The Chl content in the OX plants was slightly higher than that in the WT plants. Meanwhile, four Anti lines exhibited lower Chl b and total Chl contents (Fig. 3D, E). Moreover, the Chl content corresponded to the expression level of *PGL* (Fig. 3F). The *PGL* expression was significantly down-regulated in all four Anti lines. Consequently, the Anti lines showed the *pgl* phenotype with low Chl b and total Chl contents (Fig. 3C–F). The Chl content did not increase sharply based on the expression of *PGL* in the OX lines. In summary, the results suggest that the premature termination of the *OsCAO1* translation caused by a point mutation is responsible for the *pgl* phenotype.

### *PGL* expression pattern and subcellular localization

**Fig. 4.**
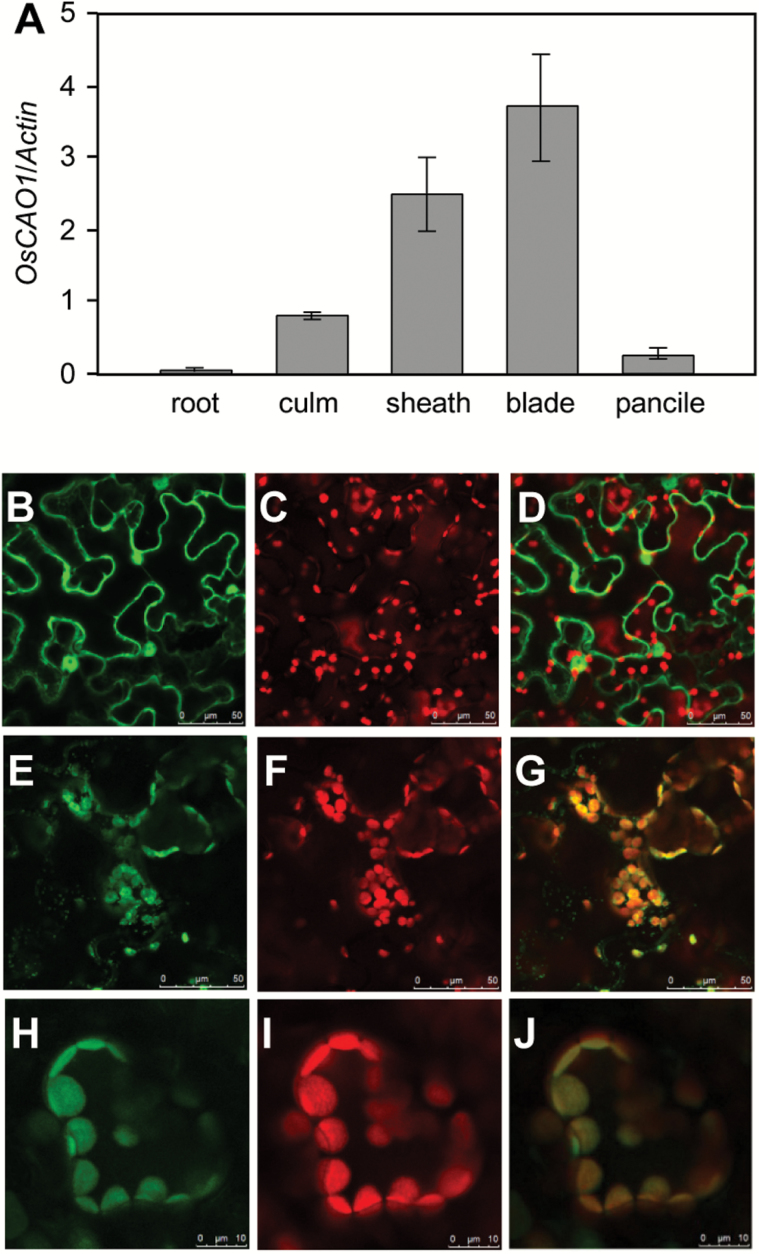
The expression pattern and sub-cellular localization of OsCAO1 in rice. (A) qRT-PCR analysis of *PGL* in rice. Total RNA was isolated from the root, culm, leaf, sheath, flag leaf and panicle. Rice *Actin1* was used as an internal control. The mean and SD values were obtained from three biological replicates. (B–J) Subcellular localization of OsCAO1 and its truncated allele. Tobacco cells transformed with: (E–G) 5S::OsCAO1-GFP; (H–J) *35S::OsCAO1* ^*pgl*^ *-GFP*; or (B–D) empty vector (*35S::GFP*) as control. (B, E, H) GFP fluorescence images; (C, F, I) chloroplast autofluorescence images; (D, G, J) overlays of GFP fluorescence and chloroplast autofluorescence images.

qRT-PCR was performed using *PGL*-specific primers to determine the expression pattern of *PGL* in rice (Supplementary Table S7). *PGL* was expressed in the culm, sheath, blade and panicle, but not the root. The expression level was highest in the blade (15-fold higher than in the panicle). These results correlated well with the level of Chl synthesis in these organs (Fig. 4A), and were also consistent with those reported previously (Lee *et al.*, 2005). Chl was synthesized in the leaf, culm, sheath, and panicle but not in the root. Furthermore, the leaf and the sheath are the major Chl synthesis organs, suggesting that *PGL* may be closely related to Chl synthesis and perhaps is one of the most important components in this pathway.

The localization of the homologous CAO of several organisms and the predication of bioinformatics suggested that OsCAO1 is most likely localized in the chloroplast. The transient expression of fluorescent protein in tobacco (*Nicotiana benthamiana*) was used to determine the subcellular localization of PGL and confirm this prediction. Subsequently, *35S::PGL-GFP* and *35S::GFP* were introduced into the epidermal cells of tobacco through *Agrobacterium* infection. GFP fluorescence was observed using a confocal laser scanning microscope. The GFP fluorescence signal was found only in the chloroplast in cells transformed with *35S::PGL-GFP* (Fig. 4E–G). In contrast, the GFP fluorescence signal was present throughout the nucleus and the cytoplasm in the cells transformed with *35S::GFP*; however, no signal overlapped between the GFP and the chloroplast (Fig. 4B–D). These results indicate that the OsCAO1 protein was localized to the chloroplast, providing further evidence for its role in Chl synthesis.

The truncated protein OsCAO1^*pgl*^ fused with GFP (*35S::OsCAO1* ^*pgl*^ *-GFP*) was also transformed into tobacco to explore whether the premature translational termination of OsCAO1 in *pgl* affects OsCAO1 localization and results in the pale-green phenotype. The results showed that OsCAO1^*pgl*^ was also localized to the chloroplast (Fig. 4H–J). Since both OsCAO1 and OsCAO1^*pgl*^ were localized to the chloroplast, the chloroplast localization signal of OsCAO1 likely exists in the N-terminus.

### *PGL* required for light-dependent Chl synthesis

**Fig. 5.**
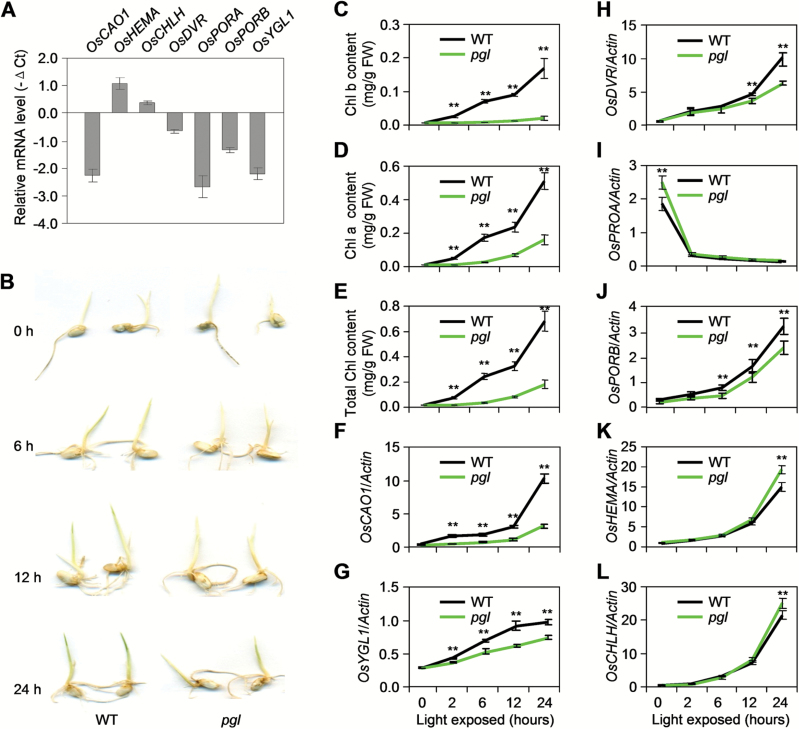
Expression levels of Chl synthesis-associated genes in *pgl*. (A) Changes in transcript levels of Chl synthesis-associated genes in WT and *pgl* leaves. (B) Comparison of greening speed in WT and *pgl* etiolated seedlings that were exposed to light (200 μmol m^−2^s^−1^) for 0–24h. (C–E) Chl content in the WT and *pgl* seedlings during greening – this analysis was performed twice with similar results: Chl b content (C); Chl a content (D); total Chl content (E). (F–L) Changes in transcript levels of Chl synthesis-associated genes in WT and *pgl* seedlings during greening: (F) *OsCAO1*; (G) *OsYGL1*, Chl synthase (LOC_Os05g28200); (H) *OsDVR*, 3,8-divinyl chlorophyllide a 8-vinyl reductase (LOC_Os03g22780); (I) *OsPORA* and (J) *OsPORB*, two protochlorophyllide oxidoreductases (LOC_Os04g58200 and LOC_Os10g35370); (K) *OsHEMA* encodes glutamyl-tRNA reductase (LOC_Os10g35840); and (L) *OsCHLH*, Mg-chelatase H subunit (LOC_Os03g20700). Mean and SD values in qRT-PCR analysis were obtained in one experiment with three biological replicates.*, *P*<0.05; **, *P*<0.01 (Student’s *t*-test).

The expression levels of *PGL* and other Chl synthesis-associated genes (i.e. *OsHEMA*, *OsCHLH*, *OsDVR*, *OsPORA*, *OsPORB* and *OsYGL1*) in the mature leaves were examined to understand the molecular basis of *PGL* involvement in Chl synthesis (Fig. 5A; Supplementary Fig. S1). The *PGL* expression level in *pgl* was significantly reduced to one-sixth of that in WT. Interestingly, the genes that function in the initial steps of Chl synthesis (i.e. *OsHEMA* and *OsCHLH*) were weakly accumulated in *pgl*, and the genes in the latter synthesis steps were down-regulated. For example, *OsPORA*, *OsPORB* and *OsYGL1* are essential for the synthesis of Chl a and its precursor. Their expression levels in *pgl* were only 15.6%, 39.1% and 21.6% of those in WT. The reduced expression levels of these genes may be responsible for the reduced Chl content in *pgl*.

The WT and *pgl* etiolated seedlings were compared during light-dependent leaf greening to further explore the contribution of *PGL* to chlorophyll synthesis. The WT etiolated seedlings quickly turned green after being exposed to light, whereas the *pgl* seedlings remained albino after 24h of light exposure (Fig. 5B). Chl b synthesis was almost entirely inhibited in the *pgl* plants (Fig. 5C). Similarly, Chl a synthesis in the *pgl* plants lagged behind that in the WT plants (Fig. 5D). As a result, the *pgl* plants presented with a lower total Chl content than the WT plants and also had pale-green leaves (Fig. 5E). These results suggest that *PGL* is essential for light-dependent accumulation of high levels of Chl. The expression of the Chl synthesis-associated genes was also detected during greening (Fig. 5F–L). The *PGL* transcript levels were steadily up-regulated under lighting when 6-day-old WT etiolated seedlings were exposed to light for 24h. In addition to *OsCAO1*, *OsHEMA*, *OsCHLH*, *OsDVR*, *OsPORB* and *OsYGL1* expression levels increased in WT plants after illumination, indicating that these genes are required for the light-dependent Chl synthesis during greening of etiolated plants. Only *OsPORA* showed no light-induced gene expression, indicating that it is not essential for this process. This is consistent with that of a previous study (Sakuraba *et al*., 2013). The expression of these genes was also measured in *pgl* plants. Compared with that observed in the WT plants, *OsHEMA* and *OsCHLH* expression levels were only mildly increased in the *pgl* plants, whereas the expression levels of *OsDVR*, *OsPORB* and *OsYGL1* that are critical for Chl synthesis, were suppressed in the *pgl* plants. A very weak increase in the *OsPORA* expression level in the *pgl* plants was also found. This increase may be related to the fact that *OsPORA* does not appear to be required for greening. These results indicate that OsCAO1 is essential not only for Chl b synthesis but also for the accumulation of high levels of Chl a by regulating key genes in Chl synthesis.

### Leaf senescence exacerbated in *pgl*

**Fig. 6.**
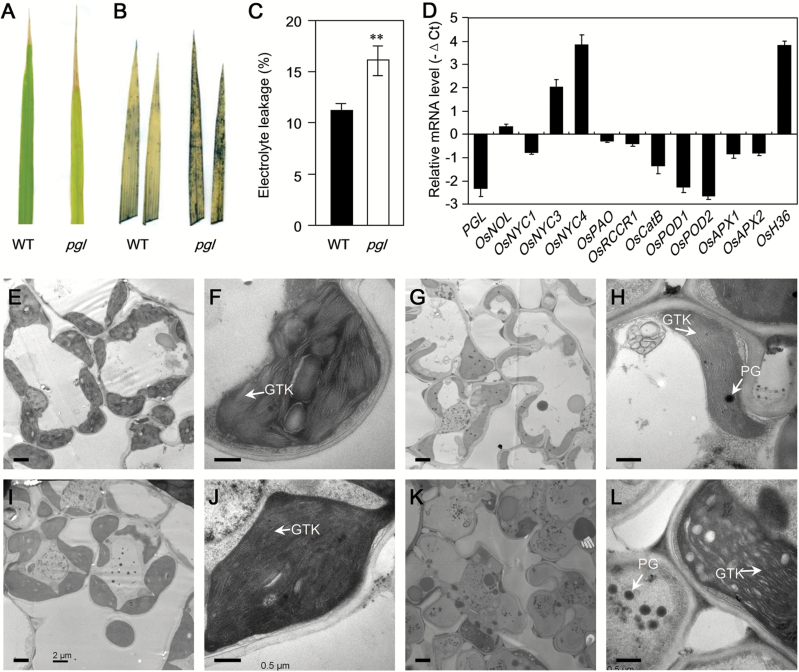
*pgl* leaves exhibit severe senescent phenotype under natural conditions. (A) Naturally senescent leaves (flag leaves) of WT and *pgl* 20 d after flowering. (B) Accumulation of superoxide anion radicals (O_2_^−^) in naturally senescent leaves, visualized by staining with NBT. (C) Electrolyte leakage in naturally senescent leaves. (D) Changes in transcript levels of senescence-associated genes in the leaves of WT and *pgl* at the mature stage. (E–L) Transmission electron microscopy (TEM) analysis of chloroplasts in *pgl* leaves. TEM analysis of the leaves of WT (E, F) and *pgl* (I, J) at the seedling stage. The samples were obtained at 20 d in the paddy field. TEM analysis of the flag leaves in the WT (G, H) and *pgl* (K, L) at the mature stage. The samples were obtained 20 d after flowering in the paddy field. *OsNOL* (LOC_Os03g45194) and *OsNYC1* (LOC_Os01g12710), two short-chain dehydrogenase/reductases, represent Chl b reductases; *OsNYC3*, α/β hydrolase-fold family protein (LOC_Os06g24730); *OsNYC4*, THYLKOID FORMATION1, chloroplast precursor (LOC_Os07g37250); *OsPAO*, pheophorbide a oxygenase (LOC_Os03g05310); *OsRCCR1*, red chlorophyll catabolite reductase (LOC_Os10g25030); *OsCatB*, catalase (LOC_Os06g51150); *OsPOD1* (LOC_Os01g22370) and *OsPOD2* (LOC_Os03g22010), two peroxidases; *OsAPX1* (LOC_Os03g17690) and *OsAPX2* (LOC_Os07g49400), two ascorbate peroxidases; OsH36, aminotransferase, senescence-induced protein (LOC_Os05g39770). GTK, grana thylakoid; PG, plastoglobule. Bar, 2 μm (E, I, G, K); bar, 0.5 μm (F, J, H, L). *, *P*<0.05, **, *P*<0.01 (Student’s *t*-test).

The *pgl* plants exhibited a pale-green leaf in the paddy field and presented a severe withered phenotype in the leaf tip (Fig. 6A). Leaf variegation or necrotic lesions were reported to result from ROS accumulation and usually resulted in cell apoptosis (Jiang *et al.*, 2011). The ROS production in the flag leaves of the WT and *pgl* plants was detected using NBT and DAB, respectively. More hydrogen peroxide accumulated in the *pgl* leaves compared with the WT leaves (Fig. 6B). The electrolyte leakage of the leaves was measured to examine whether cell death was induced in *pgl*. The electrolyte leakage in *pgl* was significantly higher than that in WT, suggesting that the *pgl* plants lost more membrane integrity during development compared with the WT plants (Fig. 6C). The expression levels of Chl degradation-associated genes (i.e. *OsNOL*, *OsNYC1*, *OsNYC3*, *OsNYC4*, *OsPAO* and *OsRCCR1*) and senescence-associated genes (i.e. *OsCatB*, *OsPOD1*, *OsPOD2*, *OsAPX1*, *OsAPX2* and *Osh36*) in mature leaves of the WT and *pgl* were compared in order to understand further the molecular basis for the early senescence phenotype (Fig. 6D). In *pgl*, *OsNYC1*, *OsPAO1* and *OsRCCR1* expression levels were slightly down-regulated, whereas *OsNOL*, *OsNYC3* and *OsNYC4* expression was up-regulated compared with that of the WT plants. The *OsNYC3* and *OsNYC4* expression levels in the *pgl* plants reached up to 4-fold and 15-fold of those in the WT, respectively. The genes associated with ROS scavenging (i.e. *OsCatB*, *OsPOD1*, *OsPOD2*, *OsAPX1* and *OsAPX2*) were all down-regulated in *pgl* compared with the level in WT (Fig. 6D). Moreover, *Osh36*, which is a senescence-inducible gene (Lee *et al.*, 2001), quickly accumulated in the *pgl* plants, reaching up to 15 times higher than that in WT. This result suggests that premature senescence occurs in *pgl*.

The TEM analysis was conducted to reveal the chloroplast morphology and structure and to understand the senescence process better. The LHC complexes included Chl b and LHC proteins, which play important roles in the stabilization of the thylakoid membranes. Few cellular differences were observed between the WT and *pgl* plants during the seedling stage (Fig. 6E, I). Compared with that in the WT plants, the grana thylakoid (GTK) in the *pgl* plants had a disorderly arrangement in the chloroplast. Furthermore, thylakoid stacking was indistinct (Fig. 6F, J). These results suggest that OsCAO1 is essential for thylakoid development. Substantial differences in the leaf cells were found at the mature stage. Accordingly, there was a large decrease in the number of chloroplasts in the *pgl* plants compared with that in the WT plants (Fig. 6G, K). Chloroplast degradation is one of the signs of senescence. Most chloroplasts in the *pgl* plants were degraded, whereas no obvious sign of chloroplast degradation was observed in the WT plants. Furthermore, the grana lamellae were arranged in an orderly manner and uniformly distributed (Fig. 6H, L). These results suggest that *pgl* exhibits a more serious senescence phenotype than WT under natural conditions in the paddy field.

**Fig. 7.**
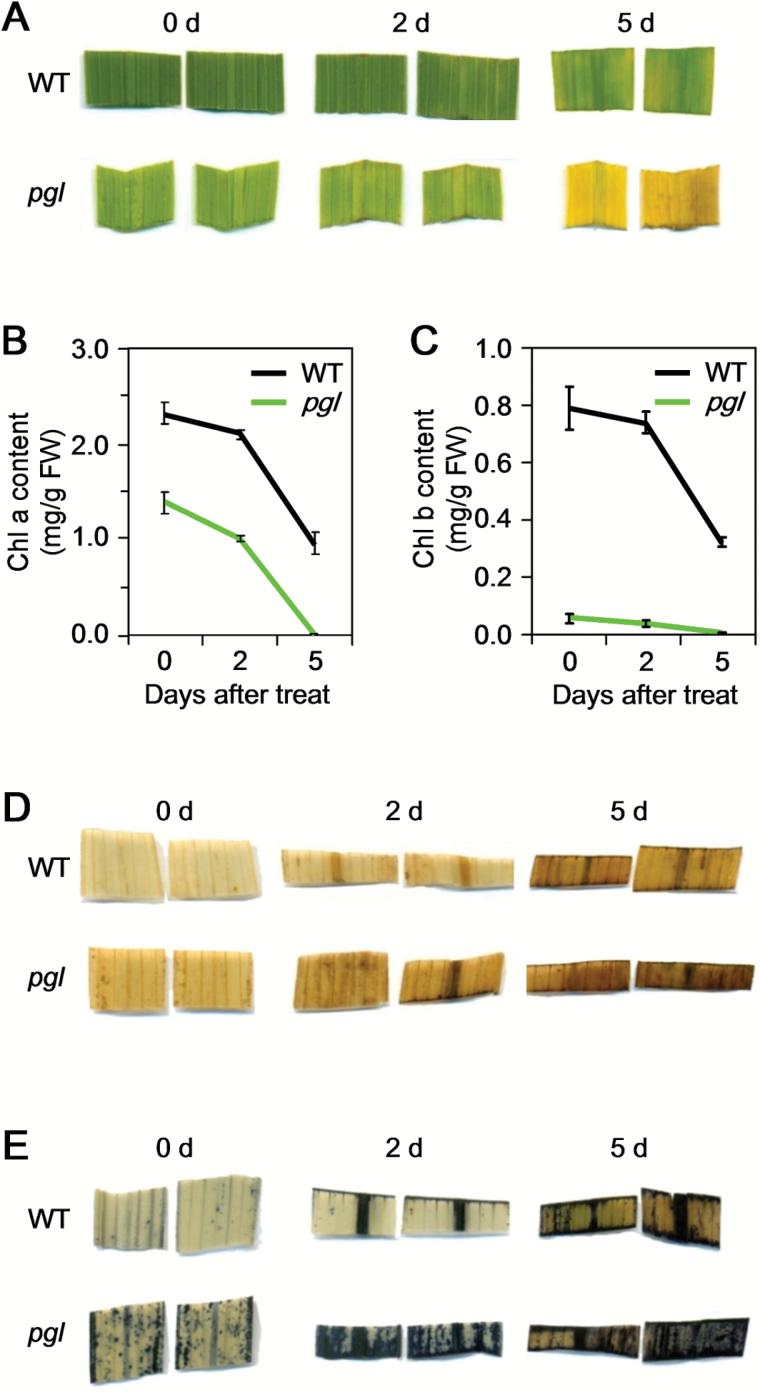
Dark-induced senescence in *pgl*. (A) WT and *pgl* leaves were incubated in the dark for 0, 2, or 5 d. (B, C) Change of Chl contents: Chl a (B) and Chl b (C), over time in *pgl* during dark incubation. (D–E) ROS accumulation in *pgl* during dark incubation. H_2_O_2_ and O_2_^−^ were detected by staining with DAB (D) and NBT (E), respectively. Mean and SD values were obtained in one experiment with three biological replicates.

Dark-induced senescence was also examined in addition to natural senescence. The detached leaves from the *pgl* and WT plants were kept in the dark at 28°C. Within five days, the leaves from the *pgl* plants turned completely yellow, and their Chl a and Chl b contents sharply decreased (Fig. 7A–C), whereas the leaves from the WT plants remained green and had high levels of Chl a and Chl b. The DAB and NBT stainings were much more pronounced in the *pgl* plants than that in the WT plants (Fig. 7D, E). Taken together, these results indicate that Chl b deficiency could speed up the aging process under dark-induced conditions.

### *pgl* sensitive to high temperature

**Fig. 8.**
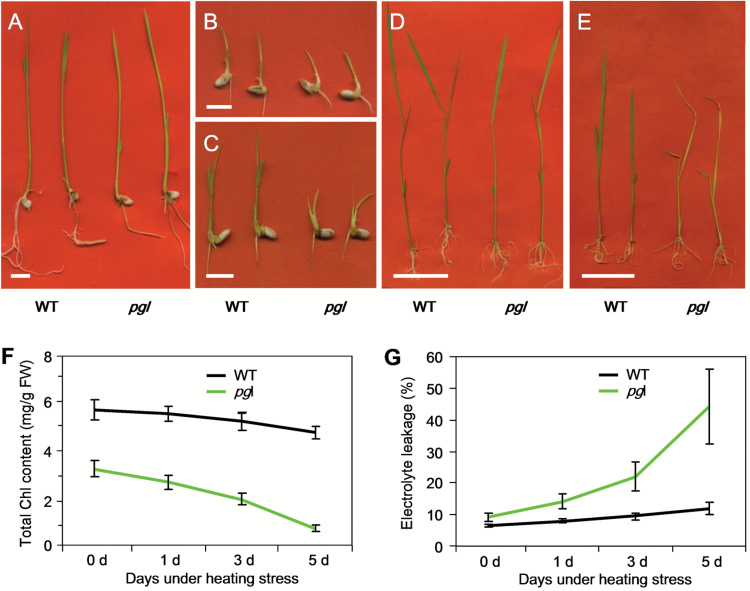
Heat sensitivity of *pgl* plants. WT and *pgl* plants were treated with 42^o^C for 16h in daytime and 35^o^C for 8h at night for heat stress. (A) WT and *pgl* seeds were germinated and grown in normal conditions, 32/25^o^C day/night temperature regime as control. (B, C) WT and *pgl* seeds were germinated and grown with heat stress for 7 d (B) and 14 d (C). (D, E) WT and *pgl* seeds were first germinated and then: grown for 1 week in normal conditions and then subjected to high temperatures for another week (E), or; were continuously grown under normal conditions as a control (D). (F) The change in total Chl content in response to heat stress for 0, 1, 3 and 5 d. (G) the change of electrolyte leakage in response to heat stress for 0, 1, 3 and 5 d. Bar, 1cm (A–C); bar, 5cm (D, E).

Temperature is one of the most important factors influencing the growth and development of plants. A continuously high temperature will accelerate rice aging. The *pgl* and WT plants grew normally under normal conditions except for the pale-green leaf phenotype associated with *pgl* (Fig. 8A). After three-day incubation at high temperature, the shoot growth was severely restricted in the *pgl* plants compared with the WT plants (Fig. 8B). By two weeks, the *pgl* plants showed obvious aging characteristics in the decayed tip on the shoot (Fig. 8C). Moreover, if the seedlings were first grown for one week at normal temperature and subsequently transferred to high temperature for another week, the *pgl* leaves rapidly wilted and scorched, whereas growth was not blocked and no aging occurred in the WT plants (Fig. 8E). As a control, the seedlings were continuously grown under normal conditions (Fig. 8D). The Chl content and the electrolyte leakage of the seedlings under heat stress were also measured. The Chl content was reduced, and the electrolyte leakage was increased in response to heat stress time (Fig. 8F, 8G). These results indicate that high temperature accelerates *pgl* aging. Accordingly, OsCAO1 or Chl b is suggested to play important roles in resisting high temperature stress.

### *PGL* affects the ROS scavenging system under low light

**Fig. 9.**
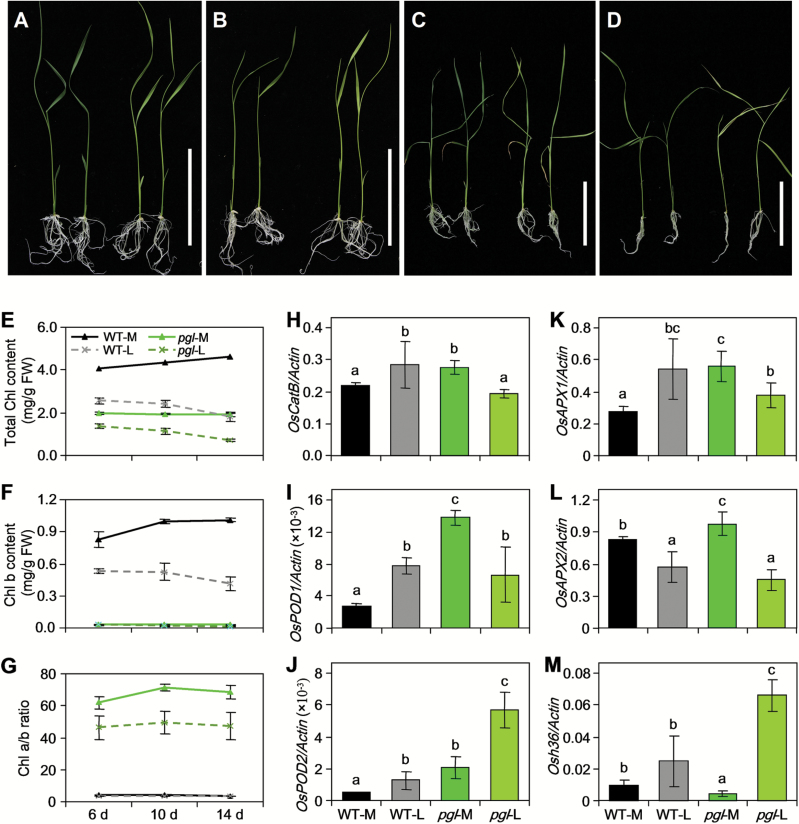
Low light treatment for WT and *pgl*. (A–D) The seedling phenotype of WT (left) and *pgl* (right) under moderate (150 μmol m^−2^ s^−1^) and low light (30 μmol m^−2^ s^−1^): for 6 d (A) and 14 d (C) under moderate treatment; for 6 d (B) and 14 d (D) under low light treatment. Bar, 10cm. (E–G) The change in the total Chl content (E), Chl b content (F) and Chl a/b ratio (G) under the low light treatment for 6 d, 10 d and 14 d. (H–M) Changes in transcript levels of senescence-associated genes in WT and *pgl* seedlings under low light treatment for 14 d: *OsCatB* (H); *OsPOD1* (I); *OsPOD2* (J); *OsAPX1* (K); *OsAPX2* (L); *Osh36* (M). WT-M and *pgl*-M indicate WT and *pgl* grown under moderate light conditions; WT-L and *pgl*-L indicate WT and *pgl* grown under low light conditions. Mean and SD values in qRT-PCR analysis were obtained in one experiment with three biological replicates. Different letters indicate significant difference at the 1% level (Duncan’s multiple range test).

Light is one of the important factors in plant growth. Low light affects the activity of the enzymes in the ROS scavenging system (Wang, 2014). To further explore the effects of ROS accumulation, we raised WT and *pgl* plants under different light conditions. The WT and *pgl* plants grew slowly under low light, and their leaves became slightly pale (Fig. 9A–D). In the WT plants, the total Chl and Chl b contents sharply decreased under the low light condition compared with normal light (Fig. 9E–G). In *pgl*, the total Chl content was also reduced under the low light condition compared with normal light (Fig. 9E), but low light had no obvious effect on the Chl b content (Fig. 9F). Subsequently, the Chl a/b ratio of the *pgl* plants was lower in low light than moderate light (Fig. 9G). Next, we assessed the expression levels of senescence-associated genes (i.e. *OsCatB*, *OsPOD1*, *OsPOD2*, *OsAPX1*, *OsAPX2* and *Osh36*) in leaves at low and normal light conditions (Fig. 9H–M). After 14 days’ exposure to low light, the expression levels of all of the genes associated with ROS scavenging except *OsAPX2* were slightly up-regulated in the WT plants. In the *pgl* plants, the expression of *OsPOD2* was up-regulated under the low light compared with that detected under normal light conditions; the other genes were down-regulated under low light compared with normal light conditions. Since the ROS scavenging system is damaged in *pgl*, *Osh36* had the highest expression level under low light conditions, suggesting that *PGL* also affects the ROS scavenging system under low light.

## Discussion

### PGL essential for Chl synthesis in rice

The role of CAO in Chl b synthesis in *Arabidopsis thaliana* has been reported previously (Espineda *et al.*, 1999). Two homologous genes of *CAO*, *OsCAO1* and *OsCAO2*, were found in the rice genome (Lee *et al.*, 2005). *OsCAO1* (i.e. *PGL*) is believed to catalyze the Chl a to Chl b conversion process in rice. The results of this study show that *PGL* encodes a chloroplast-localized protein involved in light-dependent Chl synthesis and is strongly expressed in the chlorenchyma including the culm, blade, sheath and panicle; its expression pattern correlated well with that of Chl synthesis. The premature termination of PGL resulted in the *pgl* phenotype and a lower Chl b content, which could be rescued by introducing a functional *PGL* fragment. The Chl content was substantially decreased in transgenic plants expressing anti-sense *PGL*, whereas the plants overexpressing *PGL* showed a slight increase in Chl b. Taken together, these results indicate that *PGL* is required for the Chl b biosynthesis in rice.

The mutation or inactivation of enzymes involved in the metabolic pathway of the Chl synthesis usually results in the accumulation of their respective substrates in plants (Mock and Grimm, 1997; Wu *et al.*, 2007). Chl b appears to be synthesized by the oxidation of the methyl group of Chl a to a formyl group. However, in the *pgl* plants, the Chl a content was decreased to 60% that of the WT plants. Chl a deficiency in the anti-sense transgenic plants and accumulation in *PGL*-overexpressed plants were also observed. The Chl synthesis-associated genes were further divided into two classes. Accordingly, the genes involved in the early steps (i.e. *OsHEMA* and *OsCHLH*) were up-regulated, and those involved in the later steps (i.e. *OsDVR*, *OsPORB*, *OsYGL1* and *OsCAO1* itself) were down-regulated in the *pgl* plants, suggesting that PGL may be involved in regulating Chl a synthesis in rice. Chl b deficiency promoted the expression of the Chl synthesis-related genes, especially to those involved in the early synthesis steps.

### Chl b deficiency accelerates leaf senescence in rice

Both *OsCAO1* over-expression and mutation in *NYC1* (Chl b reductase) can cause Chl b accumulation in plants and lead to a delayed senescence phenotype (Kusaba *et al.*, 2007; Sakuraba *et al.*, 2012). In contrast, a shortage of Chl b can lead to premature aging. In our study, *pgl* plants exhibited a disorganized ultra microstructure in the chloroplasts (Fig. 6E, L). Chl b existed in the light-harvesting Chl a/b-protein complex (LHCP). The LHCPs were localized to the thylakoid membrane. The LHCPs in PS II (LHC II) were encoded by the *Lhcb* gene families. Chl b is important for the stability of the LHCP (Bellemare *et al.*, 1982). *Lhcb1* and *Lhcb4* are extremely important LHC II members, and the expressions of both were reduced in the *pgl* plants (Fig. 1F, G). LHC II is predominantly localized in the grana, which is the stacking region of the thylakoid membrane, and is thought to play an important role in grana formation (Allen and Forsberg, 2001). Moreover, the presence of more PGs in the *pgl* plants compared with the WT plants is a good indicator of senescence.

In addition, in the *pgl* plants, more severe Chl degradation occurred than in the WT plants, a clear indication of senescence. The Chl content was rapidly degraded in *pgl* in dark-induced senescence. Compared with the expression levels in the WT plants, in the *pgl*, plants, *OsNYC1*, *OsPAO* and *OsRCCR* expression levels were weakly down-regulated, but *OsNYC3* and *OsNYC4* expression levels were rapidly up-regulated more than 5-fold and 16-fold, respectively (Fig. 6D). Both *OsNYC3* and *OsNYC4* play a role in regulating the Chl-protein complex degradation during leaf senescence (Morita *et al.*, 2009; Yamatani *et al.*, 2013).

A greater accumulation of ROS in the *pgl* plants compared with the WT plants can also explain why the *pgl* plants exhibited early senescence. Under normal physiological conditions, cells control ROS levels by balancing the generation of ROS with their elimination by the ROS scavenging system. In *pgl*, the scavenging system is weakened. All of the genes involved in ROS elimination – *OsCatB*, *OsPOD1*, *OsPOD2*, *OsAPX1* and *OsAPX2* – were down-regulated in *pgl*. In both naturally and dark-induced senescent plants, two detected ROS species (hydrogen peroxide and superoxide anion radicals) accumulated in the *pgl* plants (Fig. 6B; Fig. 7B, C). Oxidative damage initiated by ROS is a major contributor to the functional decline that is characteristic of aging. *Osh36*, which is expressed exclusively during senescence, was used as a molecular mark for senescence, and was obviously increased in the *pgl* plants compared with the WT plants. Excessive ROS can induce apoptosis. The electrolyte leakage in the *pgl* plants was significantly higher than that in the WT plants (Fig. 6C). Furthermore, environmental stresses may induce the senescence process because of some sources of ROS formation. The *pgl* plants that were either germinated or transplanted at a high temperature exhibited premature aging and withering within a few days (Fig. 8C, E). The high temperature sensitivity may result from the ROS accumulation in the Chl b-deficient mutants. Moreover, the *pgl* mutation can affect the increasing expression of genes associated ROS elimination under low light condition. Taken together, these results suggest that more ROS accumulates in *pgl* plants than in WT plants, potentially by affecting the ROS scavenging system.

### OsCAO1 disruption indirectly affects rice yield and quality

The leaf is the main photosynthetic apparatus and accounts for 90–95% of the dry matter in rice plants (Wu *et al.*, 2007). In this study, the *pgl* plants exhibited pale-green leaves and reduced Chl content throughout their life cycle (Fig. 1A–D). The TEM analysis revealed that the thylakoids were abnormal in the *pgl* plants (Fig. 6E–L). The thylakoid is an important place for photosynthesis; thus, it is not surprising that the photosynthesis rate is decreased in *pgl*. The prediction that decreasing photosynthesis would lead to yield decreases seemed straightforward; however, the yield decline may be regulated by several factors including both the source and the sink. A previous study found that *pgl* had a lower source (photosynthesis) than WT (Fig. 1E). However, except for the seed-setting rate, the yield traits were unaffected by this mutation (Fig. 2C; Table S1). Two different aspects of rice growth contribute to this finding. First, decreased photosynthesis results in a lack of biomass production, which can lead to an inadequate supply of nutrients for the rice grain at the filling stage (Long *et al.*, 2013). Second, the senescence in *pgl* at a later stage of rice growth was more severe than that in WT, which has a substantial influence on grain filling and further affected rice yield. In addition, abnormal grain filling causes insufficiently filled endosperm storage in the seeds and chalkiness (Lin *et al.*, 2002), which could explain why the *pgl* plants had a higher CD than the WT plants. The GC is one of the key chemical characteristics of starch in the endosperm that plays an important role in the eating and cooking qualities of rice (Fan *et al.*, 2005). In this study, the *OsCAO1* mutation resulted in a large change in the GC of the rice grain. It was speculated that the photosynthetic assimilates from *pgl* leaves were limited during the grain-filling stage, thereby affecting the grain development.

*PGL* encodes a chlorophyllide a oxygenase (i.e. OsCAO1) with functions corresponding to AtCAO. The results of this study suggest that OsCAO1 is involved in several aspects of the metabolic process. OsCAO1 was considered the only enzyme responsible for Chl b formation in rice, similar to AtCAO in *A. thaliana*. However, OsCAO1 affected Chl a synthesis by regulating the expression levels of the Chl synthesis-associated genes (e.g., *OsDVR*, *OsPORA*, *OsPORB* and *OsYGL1*). The evidence suggests that OsCAO1 also plays important roles in Chl degradation and ROS scavenging to regulate both natural and induced rice senescence.

## Supplementary data

Supplementary data are available at *JXB* online.

Fig. S1. The pathway of chlorophyll biosynthesis in plant.

Table S1. Pigment contents in leaves of wild-type and *pgl.*

Table S2. Grain yield traits in wild-type and *pgl.*

Table S3. Rice quality-related traits in wild-type and *pgl.*

Table S4. Segregation ratio of reciprocal crosses between *pgl* and *indica* varieties.

Table S5. Molecular markers (primers) used for mapping.

Table S6. Primers used for vector construction.

Table S7. Primers used for qRT-PCR.

Supplementary Data
