# Supplementary material for: PGL, encoding chlorophyllide a oxygenase 1, impacts leaf senescence and indirectly affects grain yield and quality in rice
Source: J Exp Bot. 2015 Dec 25;67(5):1297–310. doi: 10.1093/jxb/erv529 (PMC4762379; doi:10.1093/jxb/erv529)
Supplement: Supplementary Data [file supp_67_5_1297__index.html]

 PGL, encoding chlorophyllide a oxygenase 1, impacts leaf senescence and indirectly affects grain yield and quality in rice — PGL, encoding chlorophyllide a oxygenase 1, impacts leaf senescence and indirectly affects grain yield and quality in rice — Supplementary Data 

# *PGL*, encoding chlorophyllide a oxygenase 1, impacts leaf senescence and indirectly affects grain yield and quality in rice

## Supplementary Data

Data files

- Supplementary\_figure\_S1\_Tables\_S1\_S7.pdf - Supplementary Data
