# Supplementary material for: PGL, encoding chlorophyllide a oxygenase 1, impacts leaf senescence and indirectly affects grain yield and quality in rice
Source: J Exp Bot. 2015 Dec 25;67(5):1297–310. doi: 10.1093/jxb/erv529 (PMC4762379; doi:10.1093/jxb/erv529)
Supplement: Supplementary Data [file supp_erv529_Supplementary_figure_S1_Tables_S1_S7.pdf]

Glutamic acid

↓

Glutamyl-tRNA

↓ HEMA *(OsHEMA)*

Glutamate-1-1semialdehyde

↓ GSA

5-Aminolevulinic acid

↓ HEMB

Porphobilinogen

↓ HEMC

Hydroxymethylbilane

↓ HEMD

Uroporphyrinogen III

↓ HEME

Coproporphyrinogen III

↓ HEMF

Protoporphyrinogen IX

↓ HEMG

Protoporphyrin IX

↓ CHLH/D/I *(OsCHLH)*

Mg-Protoporphyrin IX

↓ CHLM

Mg-Protoporphyrin IX monomethyl ester

↓ CRD

Divinyl protochlorophylide a

↓ PORA/B/C *(OsPORA/B)*

Divinyl chlorophyllide a

↓ DVR *(OsDVR)*

Monomethyl chlorophyllide a

↓ CHLG/P *(OsYgl)*

Chlorophyll a

↓ CAO *(OsCAO1)*

Chlorophyll b

Fig. S1

Table S1 Pigment contents in leaves of wild-type and *pgl*

	Seedling stage		Tillering stage	
	Wild-type	*pgl*	Wild-type	*pgl*
Chl a (mg/g)	4.02±0.175	2.73±0.066	2.33±0.072	1.39±0.001
Chl b (mg/g)	1.19±0.014	0.13±0.002	0.79±0.083	0.05±0.006
car (mg/g)	0.89±0.049	0.723±0.04	0.50±0.060	0.33±0.012
Chl a/b	3.38	21.385	2.971	27.748

Table S2 Grain yield traits in wild-type and *pgl*

Traits	Wild-type	*pgl*
Plant height (cm)	93.75±2.217	86.5±2.645
Tillering numbers	8.5±0.58	6.3±0.50
Panicle length (cm)	20.7±0.41	20.3±1.38
No. of primary branches	13.0±0.82	13.8±1.26
No. of secondary branches	21.8±1.71	22.5±2.89
No. of grains per panicle	136.5±10.41	136.3±16.88
1000-grain weight	26.44±0.159	26.21±0.479
Seed-setting rate (%)	72.052±11.622	43.178±6.588
Grain yield per plant (g)	15.587±1.212	8.921±2.189

Table S3 Rice quality related traits between wild-type and *pgl*

Traits	Wild-type	*pgl*
Degree of chalkiness (%)	9.206±1.443	18.86±2.632
Gel consistency (mm)	73.48±3.905	48.20±0.851
Amylose content (%)	31.23 ±0.67	31.30 ±0.87
Gelatinization temperature	6	6

Table S4 Segregation ratio of reciprocal crosses between *pgl* and *indica* cultivars

Combinations	F_1		F_2		χ^2 (3:1)	*p*-value
	Wild-type	*pgl*	Wild-type	*pgl*		
pgl/ZF802	4	0	116	34	0.4356	0.5093
ZF802/*pgl*	4	0	128	38	0.3936	0.5304
pgl/NJ06	8	0	258	74	1.3012	0.254
NJ06/pgl	3	0	102	29	0.5725	0.4493
pgl/9311	5	0	154	43	1.0575	0.3038
9311/*pgl*	6	0	178	56	0.1425	0.7059

Table S5 Molecular markers (primers) for mapping in this study

Maker	Forward primers (5'-3')	Reverse primers (5'-3')
C1	ttatagaagtgcgccctaagc	agaaggttgttagccgtcca
C2	tccttggttttccttgttgc	ctcccctcctatttccgtgt
C3	tccggaggagttcatttgag	tctgggtgggctaggaagta
C4	ccttcctctgtcatcctcca	cgaaatgggtttccataccttt
C6	ccaacacaagggctcatctc	tgcgatcctgtgctaaaatg
C7	aggagattgcggggagag	atagaatcccgaaccgtcct
C8	tccatggccatacacagag	ggctcgcgctagcaattat
C9	cggttcgacctttgacaact	tgaactgctccgatttgaga
C10	tctctcggtgcatgatttga	gacccgtatattgccctgtg
M2	actttctctccatcgttgcc	aacagagttgtttcgctgcc
RM3451	cggcgagataacaattctcc	gcgtgatgatatggtatcgg
RM4771	acgttgatttcattcaggtc	acgctaactgagaaacatgg
M5	gcatgcgtacgtggatattg	gttcgtgccggtgatgtc
M6	actccttgaacagcctgcat	gtttcgatcgattcgtctcc

Table S6 Primers for vector construction in this study.

Primer	Sequence (5'-3')	Used for
Fw-OsCAO1-GFP	tctagaatgaccactgtggcatcgctgtctttgcttg	Subcellular localization of OsCAO1
Re-OsCAO1-GFP	tctagagatccactctcactttggttactgaatgg	
Re-oscao1-GFP	tctagaaccattccttcttgctcaaagcatgg	
Fw-OsCAO1-COM	gggcccgtagtcttgccctgtctcatctgcatgtcg	Genetic complementation
Re-OsCAO1-COM	gagctcaatcaggtctctttgtatagtggatgcacc	
Fw-OsCAO1-CDS	gaattcatgaccactgtggcatcgctgtctttgcttg	Over-express and anti-sense knock down
Re-OsCAO1-CDS	gaattcctatgatccactctcactttggttactgaatgg	

Table S7 Primers for qRT-PCR in this study.

Forward primer	Sequence (5'-3')	Reverse prime	Sequence (5'-3')
OsPORA-q-Fw	atggctctccaagttcag	OsPORA-q-Re	tggctcacgctaaggaac
OsPORB-q-Fw	ccgcaaggagggagcggtg	OsPORB-q-Re	ccctcttggtgctaaggccg
OsHEMA-q-Fw	cgctatttctgatgctatgggt	OsHEMA-q-Re	tcttgggtgatgattgtttgg
OsCHLH-q-Fw	aactggatgagccagaagaga	OsCHLH-q-Re	aaatgcaaaagacttgcgact
OsDVR-q-Fw	agcccaggttcatcaaggt	OsDVR-q-Re	tgatcaccctctcgaagaact
OsCAO1-q-Fw	gatccatacccgatcgacat	OsCAO1-q-Re	cgagagacatccggtagagc
OsYGL1-q-Fw	ccactggacagttgaagatgttgc	OsYGL1-q-Re	catctatatctcgatcataccagtcg
OsLhcb1-q-Fw	ccatgttctccatgttcggcttct	OsLhcb1-q-Re	taggcccaggcgttgttgttga
OsLhcb4-q-Fw	tacctgcagttcgagctggac	OsLhcb4-q-Re	aggccgaacacctcggtgta
OsCatB-q-Fw	gcttgctttctgcccagcgataat	OsCatB-q-Re	aaatagtttgggccaagacggtgc
OsPOD1-q-Fw	acgtcggggtcgccaacaac	OsPOD1-q-Re	cgaactcgtccaccgacgcc
OsPOD2-q-Fw	aggctcaactgctccagggtca	OsPOD2-q-Re	tggcaataaaccggacaagccct
OsAPX1-q-Fw	aggtgccacaaggaaagatctggt	OsAPX1-q-Re	tcagcagggctttgtcactaggaa
OsAPX2-q-Fw	tgggaagatgccacaaggagagat	OsAPX2-q-Re	tccgcagcatatttctccaccagt
OsNYC4-q-Fw	cgtctatgaccaactcatgg	OsNYC4-q-Re	tgcgtcagctctgtattgct
OsNYC1-q-Fw	ttttgagcggttcttctcaga	OsNYC1-q-Re	cctttcacaactcgcatcct
OsNOL-q-Fw	ccacgaaaggtataggatatg	OsNOL-q-Re	tcaagtcagtcaccgcagat
OsNYC3-q-Fw	tctatctaggtgccaaaggc	OsNYC3-q-Re	attctggcacctgctgtttc
OsPAO-q-Fw	aagcctccgatgttaccgaa	OsPAO-q-Re	cgagggtttccagaatttga
OsRCCR1-q-Fw	ggatcgacgattgatttcatg	OsRCCR1-q-Re	gtcgaggcgttcagaaagat
Osh36-q-Fw	cctggtgatctgaaggttgt	Osh36-q-Re	catggcaaccagtgtaaagc
Actin-q-Fw	tggcatctctcagcacattcc	Actin-q-Re	tgcacaatggatgggtcaga
